# Oral Administration of Sodium Nitrate to Metabolic Syndrome Patients Attenuates Mild Inflammatory and Oxidative Responses to Acute Exercise

**DOI:** 10.3390/antiox9070596

**Published:** 2020-07-07

**Authors:** Xavier Capó, Miguel D. Ferrer, Robert A. Olek, Eduardo Salaberry, Rafael Suau, Bartolomé Marí, Isabel Llompart, Josep A. Tur, Antoni Sureda, Antoni Pons

**Affiliations:** 1 Research Group in Community Nutrition and Oxidative Stress, University of the Balearic Islands, 07122 Palma, Spain; xavier.capo@uib.es (X.C.); miguel-david.ferrer@uib.es (M.D.F.); edusalaberry@hotmail.com (E.S.); pep.tur@uib.es (J.A.T.); antoni.sureda@uib.es (A.S.); 2 CIBEROBN (Fisiopatología de la Obesidad y la Nutrición CB12/03/30038), Instituto de Salud Carlos III, 28029 Madrid, Spain; 3 Health Research Institute of the Balearic Islands (IdISBa), 07120 Palma, Spain; isabel.llompart@ssib.es; 4 Laboratory for Physical Activity Sciences, University of the Balearic Islands, 07122 Palma, Spain; 5 Department of Athletics, Strength and Conditioning, Poznan University of Physical Education, 61-871 Poznan, Poland; robert.olek@aol.com; 6 Sports Medicine Service, Consell Insular de Mallorca, 07010 Palma, Spain; rsuau@conselldemallorca.net (R.S.); bmari@conselldemallorca.net (B.M.)

**Keywords:** exercise, inflammation, metabolic syndrome, nitrate, oxidative stress, supplementation

## Abstract

The beneficial effects of exercise for the treatment and prevention of metabolic syndrome pathologies have been related to its anti-inflammatory and antioxidant effects. Dietary nitrate supplementation is an emerging treatment strategy to alleviate the symptoms of metabolic syndrome affections and to improve vascular function. In this double-blind crossover trial, metabolic syndrome patients performed two exercise tests for 30 min at 60–70% maximal heart rate after the intake of a placebo or a nitrate-enriched beverage. Acute exercise increased the plasma concentration of TNFα, intercellular adhesion molecule ICAM1, PGE1, PGE2 and the newly detected 16-hydroxypalmitic acid (16-HPAL) in metabolic syndrome patients. The cytokine and oxylipin production by peripheral blood mononuclear cells (PBMCs) and neutrophils could be responsible for the plasma concentrations of TNFα and IL6, but not for the plasma concentration of oxylipins nor its post-exercise increase. The intake of sodium nitrate 30 min before exercise increased the concentration of nitrate and nitrite in the oral cavity and plasma and reduced the oxygen cost of exercise. Additionally, nitrate intake prevented the enhancing effects of acute exercise on the plasma concentration of TNFα, ICAM1, PGE1, PGE2 and 16-HPAL, while reducing the capabilities of PBMCs and neutrophils to produce oxylipins.

## 1. Introduction

Metabolic syndrome is a multifaceted pathology that is treated not only with pharmacological prescriptions but also with changes in lifestyle and nutritional habits. Thus, the practice of regular physical activity [1,2,3]; avoidance of sedentarism; and consumption of functional foods with beneficial effects on the metabolism, inflammation, and cardiovascular function are recommended. Exercise protects against stress conditions [4] by inducing metabolic and biochemical adaptive responses that contribute to maintaining health and counteracting illness [5]. Regular physical activity reduces the risk of several diseases, such as those related to the metabolic syndrome, cancer, anxiety, and depression [6]. Most of these diseases are directly or indirectly related to inflammation and oxidative stress processes, and the therapeutic benefits of exercise on these pathologies have been related to anti-inflammatory and anti-oxidant effects [1], amongst others. In order to attain these healthy benefits, the current recommendation is to do moderate exercise for at least 30 min a day [7]. The systemic effects of exercise are mediated through diverse molecules, some of which are secreted from the contracting muscle (myokines), contributing to the antioxidant and anti-inflammatory effects of exercise training [8]. Although secretome analyses have enabled the identification of polypeptide myokines released by a contraction in myotubes and human skeletal muscle [9,10], their physiological function, regulation, and relevance in vivo remain unclear. Other molecules that have been linked to the protective effects of exercise are oxylipins: a family of oxygenated products formed by enzymes such as cyclooxygenases (COXs), lipoxygenases (LOXs), and cytochrome P450 epoxygenase (CYP450) using fatty acids as substrates [11,12]. Circulating oxylipin concentration is typically low, but tissue production can be increased tremendously (about 10-fold) in response to stimulation [13]. It has been reported that oxylipins are able to regulate muscular adaptations to exercise and that they participate in the inflammatory or anti-inflammatory response to exercise [14,15].

Reduction of nitric oxide (NO) bioavailability plays a central role in the pathophysiology of metabolic dysfunction [16]. NO is mostly produced by nitric oxide synthases (NOS) using L-arginine as a substrate. An alternative source of NO may be dietary nitrate [17,18,19]. Nitrate from the diet is absorbed in the upper gastrointestinal tract and concentrated in saliva [20], where it is reduced to nitrite by bacterial nitrate reductases in the mouth [21]. Salivary nitrite can be reduced to NO in the acidic environment of the stomach [22] or can enter plasma [20], where several enzymes can reduce it to NO in hypoxic conditions. It has even been pointed out that nitrate could be directly reduced to nitrite and NO by tissue and circulating xanthine oxidase [23].

Therefore, dietary nitrate supplementation has been proposed as an emerging treatment strategy to alleviate symptoms of metabolic syndrome and improve vascular function [17,18,24]. Additionally, the potential use of nitrate supplements as ergogenic aids in physical performance has been evaluated: dietary nitrate supplementation reduces the oxygen cost of submaximal and maximal exercise, improves mitochondrial efficiency and contractile energetics during exercise [25,26,27], and could reduce the ATP cost of force production [28]. Reduction of the oxygen cost of exercise after dietary nitrate supplementation could be partially attributed to the decrease in the oxygen consumption in processes other than the respiratory chain, such as the enzyme activities of COXs, LOXs, NADH oxidases, xanthine oxidase, and/or NOS. In this respect, nitrite has been demonstrated to attenuate NADPH oxidase-derived superoxide generation in activated macrophages and human monocytes via a nitric oxide-dependent mechanism [29]. Evaluation of the effects of exercise and dietary nitrate on the products of these oxidases could enable the contribution of these processes to oxygen consumption to be understood. Interactions between NO and myokines in skeletal muscle during exercise are not fully elucidated, but NO production can act as a signal to activate the expression of myokines IL-6 and IL-8 [30], as well as a vascular endothelial growth factor (VEGF) A [31]. Therefore, we aimed to demonstrate the effects of dietary nitrate on the circulating concentrations of oxylipins and cytokines, as well as the contribution of peripheral blood mononuclear cells (PBMCs) and neutrophils to its production, in response to an acute bout of moderate exercise in metabolic syndrome patients.

## 2. Materials and Methods 

### 2.1. Study Subjects

A total of 14 male participants from the randomized, multicenter, clinical trial with parallel groups with metabolic syndrome (PREDIMED-Plus) were enrolled in this study. Inclusion criteria included men and women aged 55–75 years old, with a body mass index (BMI) between 27 and 40 kg/m^2^, which met at least three criteria for metabolic syndrome according to the updated harmonized criteria of the International Diabetes Federation and the American Heart Association and National Heart, Lung, and Blood Institute. Exclusion criteria were: (a) documented history of previous cardiovascular disease, (b) active cancer or a history of malignant tumors in the last five years, and (c) impossibility to follow a recommended diet or to carry out physical activity. The study was conducted after two years of PREDIMED-Plus intervention in exercised metabolic syndrome patients. All procedures were approved by the Research Ethics Committee of the Balearic Islands (reference no. 3560/17 PI) and were performed according to the guidelines laid down in the Declaration of Helsinki. All participants were informed of the purpose and the implications of the study, and informed consent was obtained from all subjects.

### 2.2. Basal Measurements 

Height was determined using a mobile anthropometer (Kawe 44444, Asperg, Germany) to the nearest millimetre, with the participant’s head placed in the Frankfurt plane. Bodyweight was determined to the nearest 0.1 kg using a digital scale (Tefal, sc9210, Rumilly, France). Participants were weighed with bare feet and wearing light underwear. Body mass index was calculated using the formula BMI = mass (kg)/squared height (m). Waist circumference (WC) was measured as the smallest horizontal girth between the costal margins and the iliac crests at minimal respiration using a non-stretchable measuring tape (Kawe 43972, Kirchner and Wilhelm GmBH Co., KG, Asperg, Germany) to the nearest 0.1 cm. All anthropometric measurements were performed by one observer to avoid inter-observer variation. Blood pressure was measured in triplicate with the participant in a seated position after resting quietly for 5 min, using a validated semi-automatic oscillometer (Omron HEM-705CP, Kyoto, Japan) [32].

### 2.3. Experimental Procedure

Subjects arrived at the laboratory at 08:30 on two separate occasions placed one week apart to perform a submaximal exercise test. The subjects arrived at the postprandial situation after the intake of a standardized breakfast avoiding nitrate-rich foods. The breakfast was consumed one hour before the first blood extraction. On each occasion, the subjects had the same standardized breakfast. Following blood sampling (pre-exercise), we determined the oral nitrate-reducing capability in each subject; after that, the subjects consumed 500 mL of an almond-based beverage: placebo (made up of 3.0% almond, 0.8% sucrose, 0.6% olive oil, and 1.8 mg/100 mL α-tocopherol acetate) (Vitalmend SL, Palma, Spain) [33] or the same beverage supplemented with sodium nitrate (16 mM) in a double-blind crossover fashion. Thirty minutes following the beverage consumption, participants performed submaximal tests at 60–70% of the participant’s maximal heart rate (HRmax), on a motorized treadmill (H/P/cosmos^®^, pulsar^®^). During the exercise test, subjects were equipped with a heart rate transmitter that recorded heart rate (HR) values. Oxygen uptake (VO_2_) and carbon dioxide production (VCO_2_) were recorded continuously using a computerized metabolic cart (Ultima series^®^, Medgraphics cardiorespiratory diagnostics, Saint Paul, MN, USA). A second venous blood sample was taken thirty minutes after the end of the exercise (post-exercise sample).

Venous blood samples were obtained from the antecubital vein of participants in suitable vacutainers with EDTA as an anticoagulant for blood cell count analyses, to obtain plasma, and to isolate PBMCs and neutrophils. 

Haematological parameters were determined in an automatic flow cytometer analyzer Technicon H2 (Bayer, Leverkusen, Germany) VCS system. C reactive protein was determined by standard procedures using a commercial clinical kit in a Technicon DAX autoanalyzer system (Bayer, Leverkusen, Germany).

### 2.4. Plasma, PBMCs, and Neutrophils Isolation

Plasma was obtained after blood centrifugation at 900× *g* for 30 min at 4 °C, and samples were stored at −80 °C until their utilization. 

PBMCs were obtained following a method previously described [33]. Blood was carefully introduced on Ficoll in a proportion of 1.5:1 and was then centrifuged at 900× *g*, at 4 °C for 30 min. The PBMCs layer was carefully removed. The plasma and the Ficoll phases were discarded and the cell precipitate was used to isolate neutrophils. The PBMCs slurry was then washed twice with PBS and centrifuged for 10 min at 1000× *g*, at 4 °C. This process was performed in duplicate with one of the samples used to obtain RNA, while the other one was used to culture PBMCs for 2 h at 37 °C.

Neutrophils were obtained from Ficoll cell precipitate using a method previously described [34]. This precipitate was suspended in PBS and centrifuged at 750× *g*, at 4 °C for 15 min, and the supernatant was then discarded. The neutrophil phase at the bottom was washed first with ammonium chloride (0.15 M) and then with phosphate buffer saline (PBS), pH 7.4. This process was performed in duplicate with one of the samples used to obtain RNA, while the other one was used to culture neutrophils for 2 h at 37 °C. 

### 2.5. Cytokine and Oxylipin Production by PBMCs and Neutrophils

The production of cytokines and oxylipins by PBMCs and neutrophils was monitored in the supernatants of these cells cultured for 2 h. PBMCs and neutrophils were isolated from 2 mL of the blood of participants in pre- and post-exercise condition after the intake of placebo or nitrate-enriched beverages. Isolated cells were then resuspended and cultured for two hours at 37 °C in 2 mL of RPMI 1640 culture medium containing 2 mM L-glutamine. Immune cells concentration on culture media was therefore the same as in blood. At the end of the two hours, samples were centrifuged at 900× *g*, for 5 min, at 4 °C and the cell-free supernatants were stored at −80 °C until cytokine, adhesion molecules and oxylipin determination. 

### 2.6. Cytokine and Adhesion Molecules Determination

Cytokine (IL6 and TNFα) determinations were performed in cell-free supernatant using individual ELISA kits (Diaclone^®^, lit for GENPROBE, Besancon, France) according to the manufacturer’s instructions. The overall intra-assay coefficient of variation was calculated to be 3.3% for TNFα and 4.4% for IL6; the calculated overall inter-assay coefficient of variation was 9.0% for TNFα and 9.1% for IL6.

Intercellular adhesion molecule 1 (ICAM1) determination was performed in cell-free supernatant using individual ELISA kits (CUSABIO^®^, Houston, TX, USA) according to the manufacturer’s instructions. The overall intra-assay coefficient of variation was calculated to be lower than 8% and the inter-assay coefficient of variation was lower than 10.0%.

### 2.7. Oral Nitrate-Reducing Capability

Participants were instructed to hold 10 mL of water in their mouth for 2 min, and the process was repeated with 10 mL of a solution of 8 mM sodium nitrate. The mouth rinse was collected into a sterile tube and centrifuged (4500 rpm, 4 °C) for 10 min. The supernatant was collected and stored at −80 °C before the measurement of nitrite and nitrate concentration as indicated below.

### 2.8. Nitrite and Nitrate Concentrations

Nitrite and nitrate concentrations were evaluated in plasma and in the mouth rinse supernatants. Plasma samples were thawed and deproteinized (200 μL of the sample, 400 μL of zinc sulphate in deionized water at 10% weight/volume and 400 μL of 1 M sodium hydroxide in deionized water) for the determination of nitrate in order to avoid scum interferences during the determination [35]. The determination of nitrate and nitrite in the mouth rinse supernatants and of nitrite in plasma were performed directly without deproteinization. Nitrate and nitrite measurements were made using a NO analyzer (NOA 280i; Sievers, GE Power and Water, Boulder, CO, USA) detecting chemiluminescence produced by the reaction between ozone and nitric oxide [36]. Nitrite was transformed into nitric oxide in a customized glass purge vessel infused with nitrogen using triiodide reagent (2.5 mL glacial acetic acid, 0.5 mL of 18 Ω deionized water and 25 mg sodium iodide) with 100 μL of anti-foaming agent at 50 °C. Nitrate was transformed into nitric oxide in the glass purge vessel using vanadium reagent (32 mg of vanadium tri-chloride, 4 mL of 1M hydrochloric acid and 500 μL of water) and 100 μL of anti-foaming agent at 95 °C [33]. This purge vessel was further connected to the NO analyzer. Nitrate and nitrite standard curves were constructed in order to determine the nitrate and nitrite concentration in plasma and in the mouth rinse supernatants.

### 2.9. Oxylipins and Free Fatty Acid Determination

Oxylipin and free polyunsaturated fatty acids were measured in plasma and in the cell-free supernatant of cultured PBMCs and neutrophils, following a modification of the method previously described [37]. Plasma and cell-free supernatant samples were first diluted 1:1 with formic acid 0.1% and centrifuged at 1200× *g* to eliminate the precipitated proteins. Free fatty acids and oxylipins were extracted and concentrated with the Strata ^®^ C-8 cartridge according to minor modification of the method previously described [38]. Briefly, the Strata ^®^ C-8 cartridge (100 mg, 55 µm, 70 Å), from Phenomenex^®^, connected to a Visiprep SPE vacuum manifold (Supelco Co., St. Louis, MO, USA), was washed with 1 mL of methanol followed by 1 mL of 0.1% formic acid solution. 1 mL of formic-deproteinized samples with internal standard solutions added was applied to the Strata^®^ C-8 columns. The columns were subsequently washed with 1 mL of 0.1% formic acid solution. All analytes were eluted with 1 mL of methanol. The eluate was evaporated using an Eppendorf Concentrator 5301^®^. The resulting residues were dissolved in 50 μL of 50% methanol and injected into the liquid chromatography-mass spectrometer/mass spectrometer MS/MS system. 

An ultra-high-performance liquid chromatography system (Ultimate 3000, ThermoFisher^®^ Scientific, Waltham, MA USA) coupled to a Q-Exactive Hybrid Quadrupole-Orbitrap mass spectrometer (ThermoFisher^®^ Scientific) operating with a heated electrospray interface (HESI) were employed and the spectra were recorded in negative mode. The analytical column was a Luna C8 (150 mm × 2.0 mm, 5 µm; Phenomenex, Torrance, CA, USA) maintained at 40 °C. The mobile phases consisted of 0.5 mM ammonium formate (pH 3.3) (A) and acetonitrile containing 0.5 mM ammonium formate (B). The stepwise linear gradient was as follows: 5% B at 0 min, 35% B at 5 min, 65% B at 15 min, 75% at 20 min, 100% B at 24 min, 10% B at 28 min, and 5% B at 29 min. The flow rate was 0.3 mL/min, and the injection volume was 10 µL. Temperatures of ion transfer capillary, spray voltage, sheath gas flow rate, auxiliary gas flow rate and S-lens RF level were set to 350 °C, 3.1 kV in negative mode, 35 arbitrary units (AU), 10 AU and 55 AU, respectively. Full scan acquisition over a range of 150–700 *m*/*z* was performed with a resolution of 70,000. During the MS/MS scans, precursors were fragmented with normalized collisional energy of 60 AU. Ions were selected for MS/MS analysis from an inclusion list in accordance with the *m*/*z* and retention time found by using each standard oxylipin and free fatty acid assayed. Xcalibur™ 4.1, Trace Finder 4.1 SP2 software were used for data processing (ThermoFisher^®^ Scientific). 

Oxylipin and free fatty acid concentrations were calculated using deuterated 20-hydroxyeicosatetraenoic acid (20-HETE-d6), deuterated Prostaglandin E2 (PGE2-d4) and deuterated Prostaglandin F2α (PGF2α-d4) as internal standards from Cayman Chemical (Ann Arbor, MI). Previously, we analyzed the individual response of each oxylipin and free fatty acid with respect to their internal standards using pure commercial-free fatty acids or oxylipins. A mixture of oxylipins, free fatty acids and deuterated internal standards at a known concentration in water was processed as indicated above for plasma or cell-free supernatants. The response factor (RF) for each oxylipin or free fatty acid was calculated with respect to the response of its deuterated internal reference compound. These RFs were used to calculate the concentration of oxylipins and free fatty acids in plasma and in cell-free supernatants. The oxylipins and free fatty acids analyzed were: 5,8,11,14-eicosatetraenoic acid (AA), 8,11,14,17-eicosatetraenoic acid (ETA), 5-hydroxy-6,8,11,14-eicosatetraenoic acid (5-HETE), 15-hydroxy-5,8,11,13-eicosatetraenoic acid (15-HETE), 20-hydroxy-5,8,11,13-eicosatetraenoic acid (20-HETE), prostaglandin E1 (PGE1), prostaglandin E2 (PGE2), 5,8,11,14,17-eicosapentaenoic acid (EPA), 15-hydroxy-5,8,11,13,17-eicosapentaenoic acid (15-HEPE), 4,7,10,13,16,19-docosahexaenoic acid (DHA), 17-hydroxy-4,7,10,13,16,19-docosahexaenoic acid (17-DoHE), resolvin D2 (RvD2), and 16-hydroxypalmitic acid (16-HPAL).

### 2.10. Statistical Analysis

A Shapiro–Wilk test was applied to identify the distribution profile of the variables. A paired sample t-test was applied when comparing two groups in which samples followed a normal distribution. A Wilcoxon test was applied when comparing two groups in which samples did not follow a normal distribution. A two-way ANOVA for repeated measures was applied when analyzing the effect of two factors and samples followed a normal distribution. When significant effects were found, a paired sample t-test was applied to determine the differences between the groups involved. A multiple Wilcoxon test with 5% false discovery rate (FDR) correction was applied when analyzing the effect of two factors and samples did not follow a normal distribution. *p* < 0.05 was considered significant unless stated otherwise.

## 3. Results

Subject characteristics and basal blood markers are presented in Table 1.

There were no differences in the duration of exercise tests or walking speed after the intake of the placebo or the nitrate-enriched beverages (Table 2); in spite of this, intake of the nitrate-enriched beverage significantly lowered heart rate, VO_2_, and VCO_2_. Thus, nitrate intake significantly reduced both total and rate of energy expenditure and energy efficiency by about 11–12% when compared to placebo intake. Nitrate intake maintained the values of the respiratory exchange ratio at the same level as a placebo, showing that nitrate supplementation maintained the same contribution of carbohydrate and lipids to the energy expenditure of exercise.

**Figure 1:**
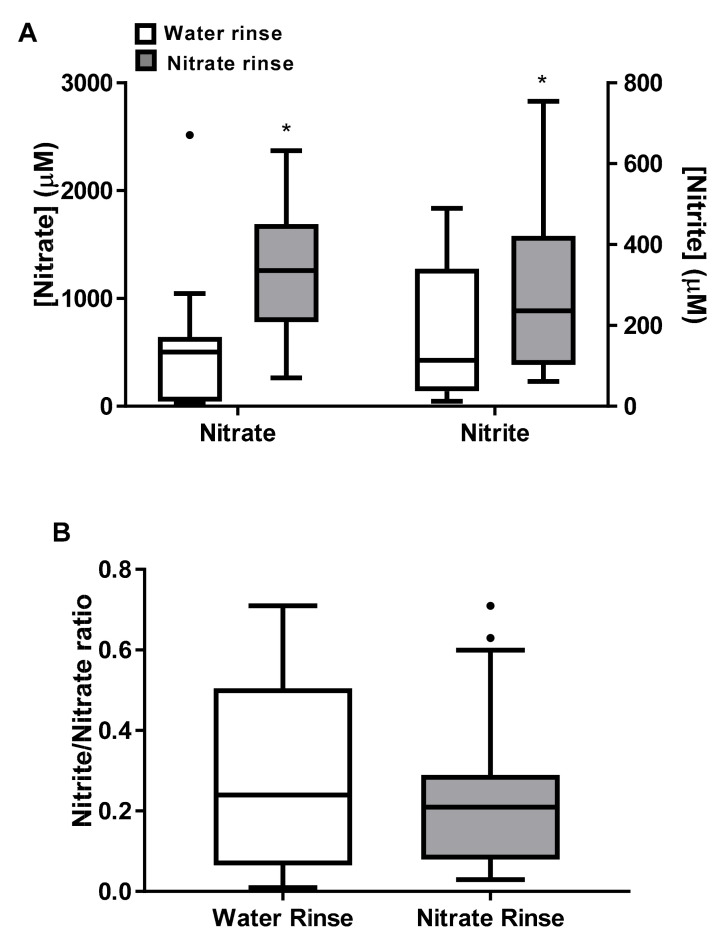
Oral nitrate-reducing capability. (**A**) Nitrate and nitrite concentrations in a mouth rinse liquid; (**B**) nitrite/nitrate ratio in a mouth rinse liquid. Participants were instructed to hold in their mouth 10 mL of water or water containing sodium nitrate (80 μmol) for 2 min and nitrate and nitrite concentration were measured in the recovered mouth rinse liquid. Results represent the median with interquartile range and outliers (●). Normality test: Shapiro–Wilk. All the parameters followed a non-normal distribution. Statistical analysis: Wilcoxon test, (*) represents significant differences vs. placebo (*p* < 0.05).

Since the possible effects of sodium nitrate intake could be related to its reduction to nitrite in the oral cavity by anaerobic bacteria, oral nitrate-reducing capability was evaluated by the determination of nitrate and nitrite concentration in a mouth rinse of sodium nitrate solution (Figure 1).

Both nitrate and nitrite concentrations were higher in the mouth rinse of sodium nitrate solution than in the mouth rinse of water, proving the nitrate-reducing capability of the oral cavity. It is remarkable that the nitrite/nitrate ratio in the mouth rinse of water was similar to the mouth rinse of sodium nitrate solution, pointing to a quick equilibrium in the reduction of nitrate to nitrite in the oral cavity. Meanwhile, the nitrite concentration attained in the liquid after mouth rinsing was at micromolar level, about 1000-fold higher than nitrite plasma concentration (nanomolar level) (Table 3), even when the rinsed liquid was originally water. An acute bout of exercise after intake of a placebo beverage did not affect the values of plasma nitrate and nitrite. However, when the same exercise was performed after intake of the nitrate supplemented beverage, plasma nitrate and nitrite concentrations significantly increased about 10- and 1.8-fold, respectively. The plasma nitrite/nitrate ratio was about 100 times lower than the nitrite/nitrate ratio in the oral cavity. Acute exercise significantly reduced the plasma nitrite/nitrate ratio only after intake of the nitrate-enriched beverage.

Acute exercise and nitrate supplementation did not influence neutrophil or PBMC counts (Table 4). Plasma markers of inflammation such as TNFα and ICAM1 increased about 1.8 and 1.3 times, respectively, after acute exercise when participants had drunk the placebo beverage (Table 4). This increase was not observed when the exercise was done after intake of the nitrate enriched beverage. Therefore, nitrate intake prevented the mild plasma inflammatory response induced by acute exercise at moderate intensity and duration in metabolic syndrome patients. No effects of nitrate consumption or acute exercise were observed in plasma IL6 or C reactive protein concentrations.

Acute exercise significantly increased PGE1, PGE2, and 16-HPAL plasma concentrations about 1.4, 3 and 2 times, respectively; however, this increase was not observed when acute exercise was performed after intake of the nitrate enriched beverage (Table 5). The 15-HEPE post-exercise plasma concentration in the placebo trial was significantly higher (about 1.7 times) than the post-exercise concentration in the nitrate trial. Nitrate intake and acute exercise did not influence the plasma concentrations of AA, ETA, 5-HETE, 15-HETE, 20-HETE, EPA, DHA, or RvD2.

Cytokine and oxylipin concentrations were measured in the extracellular media of PBMCs and neutrophils collected in basal conditions (before performing the exercise test and before the intake of the placebo or supplemented beverage) and cultured for 2 h at the same concentration as in blood (PBMC 7.5 ± 0.8 million cells in 2 mL, neutrophils 7.8 ± 0.9 million cells in 2 mL) (Table 6). The concentration of TNFα and IL6 attained in the culture medium after 2 h of incubation, as indicators of the production of these cytokines by PBMCs and neutrophils, were at the same level or higher than TNFα and IL6 plasma concentrations; however, the concentration of fatty acids and oxylipins in the culture medium after incubation of these immune cells was lower than their plasma concentrations, with the exception of 16-HPAL, which could be produced by PBMCs and neutrophils, thereby contributing to its basal plasma concentrations.

**Figure 2:**
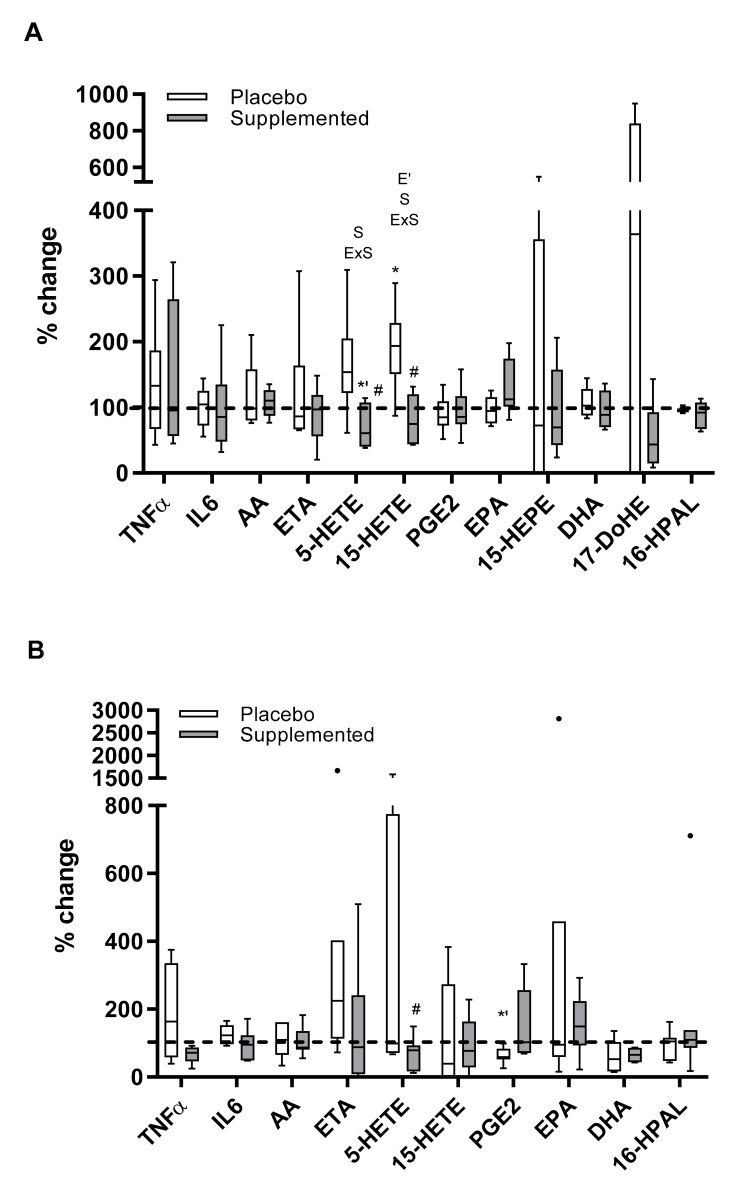
Effect of exercise and nitrate supplementation on the production of cytokines, fatty acids and oxylipins by PBMCs and neutrophils. Results represent the % change of Post-exercise production with respect to Pre-exercise production (100%, dashed line). Results are shown as median with interquartile range and outliers (●). Normality test: Shapiro–Wilk. (**A**) Peripheral blood mononuclear cells (PBMCs); AA, 5-HETE, 15-HETE, DHA, 16-HPAL, normal distribution; remaining parameters, non-normal distribution. (**B**) Neutrophils; DHA, normal distribution; remaining parameters, non-normal distribution. Statistical analysis: Multiple Wilcoxon test for not normally distributed parameters; two-way ANOVA for repeated measures in normally distributed parameters. S represents significant effect of supplementation; ExS represents significant interaction of exercise and supplementation; * represents significant differences vs. Pre-exercise (*p* < 0.05); # represents significant differences vs. Placebo (*p* < 0.05 for normally distributed parameters, *p* < 0.025 for not normally distributed parameters). E’ represents the significant effect of exercise; *’ represents significant differences vs. pre-exercise (*p* < 0.1). AA: 5,8,11,14-Eicosatetraenoic acid (Arachidonic acid); DHA: 4,7,10,13,16,19-docosahexaenoic acid; 17-DoHE: 17-Hydroxy-4,7,10,13,16,19-docosahexaenoic acid; ETA: 8,11,14,17-eicosatetraenoic acid; EPA: 5,8,11,14,17-eicosapentenoic acid; 5-HETE: 5-Hydroxyi-6,8,11,14-Eicosatetraenoic acid; 15-HETE: 15-Hydroxy-5,8,11,13-Eicosatetraenoic acid; 15-HEPE: 15-Hydroxy-5,8,11,13,17-Eicosapentaenoic acid; 16-HPAL: 16-Hydroxy-hexadecanoic acid PGE1: Prostaglandin E1; PGE2: Prostaglandin E2.

PBMCs and neutrophils could contribute to basal plasma concentrations of TNFα and IL6 but, in general, their contribution to the plasma concentrations of fatty acids and oxylipins could be scarce at the basal level. PBMC capability of TNFα, IL6, 5-HETE, 15-HETE, 15-HEPE, DHA, 17-DoHE, 16-HPAL, and PGE1 production was significantly higher when compared to neutrophils, but PGE2 production was significantly lower in PBMCs than in neutrophils. These basal capabilities were influenced by acute exercise and nitrate ingestion (Figure 2). Acute exercise significantly increased 15-HETE production by PBMCs, but significantly decreased PGE2 production capability by neutrophils. These effects induced by acute exercise were avoided by the intake of the nitrate-enriched beverage, thus maintaining pre-exercise capabilities to produce fatty acids and oxylipins by PBMCs and neutrophils. Moreover, the post-exercise production of 15-HETE and 5-HETE by PBMCs and the post-exercise production of 5-HETE by neutrophils after intake of the nitrate-enriched beverage were significantly lower, 20–34%, than after the intake of the placebo beverage.

## 4. Discussion

Regular physical activity prescription is a key point for the management and prevention of chronic diseases such as those related to the metabolic syndrome [1,2,3], most of which are directly or indirectly related to inflammation and oxidative stress processes. The therapeutic benefits of exercise on these pathologies have been related to anti-inflammatory and anti-oxidant effects [1], amongst others. Thereby, daily repetition of the metabolic and biochemical responses to each acute bout of exercise induces the beneficial adaptation to maintain a healthy status. Here we report that the practice of moderate acute exercise (60–70% of HRmax) for 30 min by metabolic syndrome patients increased TNFα and ICAM1 concentrations but maintained pre-exercise concentrations of IL6 at 30 min post-exercise. These observed changes might be interpreted as a mild pro-inflammatory response to exercise in metabolic syndrome patients. While low-to-moderate intensity aerobic exercise training favorably decreases cellular adhesion molecules (CAMs) in a variety of populations, CAMs momentarily increase immediately following high-intensity aerobic exercise [39]. This might indicate that even moderate-intensity exercise can have acute pro-inflammatory effects on metabolic syndrome patients, and some exercise-induced responses related to muscle contraction may be attenuated. More intense or longer exercise ought to be performed in order to obtain a systemic myokine response.

The systemic effects of exercise are mediated through diverse molecules, such as myokines secreted from the contracting muscle, although other compounds derived from the oxidation of polyunsaturated fatty acids, such as oxylipins, could contribute to the antioxidant and anti-inflammatory effects of exercise training [40]. Moderate acute exercise increased plasma concentration of the oxylipins PGE1 and PGE2, and the hydroxyl saturated fatty acid 16-HPAL. Prostaglandins are produced by oxidation of arachidonic acid by COXs, followed by the metabolism of endoperoxide by terminal prostaglandin synthases [41]. COX are ubiquitous enzymes that are considered pro-inflammatory because their inhibition produces anti-inflammatory effects [42]. Increased plasma concentrations of PGE1 and PGE2 after moderate acute exercise probably reflect an exercise-enhanced operation of COXs. It has been shown that football training increased PGE1 plasma concentrations and intense exercise increased PGE2; in addition, exercise has been demonstrated to prime PBMCs to produce PGE1, PGE2, and RvD1 in well-trained footballers [40]. Here, we have found that metabolic syndrome patients also respond with increased PGE1 and PGE2 plasma concentration to moderate short-term exercise. The basal production of PGE1 and PGE2 by PBMCs and neutrophils does not seem to allow the observed basal plasma concentrations of PGE1 and PGE2 to be attained; in addition, the increased production of PGE1 and PGE2 by PBMCs and neutrophils after exercise cannot influence the plasma concentration of these oxylipins in metabolic syndrome patients. Therefore, the post-exercise plasma concentration of these prostaglandins probably reflects their secretion by tissues other than immune cells. The contribution of muscle COXs to prostaglandin plasma concentration has already been described [43]; in fact, prostaglandins are synthesized by skeletal muscle and regulate muscle responses and adaptations to exercise. Acute resistance exercise increased muscle COX protein levels and activity in healthy young athletes [44]; similarly, here we report increased PG plasma concentration after moderate acute exercise in aged metabolic syndrome patients. The changes in plasma concentration of 16-HPAL are reported for the first time. ω-Hydroxyacids of saturated and unsaturated fatty acids are produced by cytochrome P450-dependent mechanisms in both plants and in mammals [45]. The ω-oxidation of saturated fatty acids mainly occurs in liver and kidney cells [46]. In addition, the presence of ω-hydroxyacids has been described in meibum, a lipid mixture produced by specialized sebaceous glands along the edge of the eyelids [47]. The function and properties of 16-HPAL are not yet described, but their plasma concentration responds to acute exercise and both PBMCs and neutrophils are able to produce it.

These pro-inflammatory responses to moderate acute exercise in metabolic syndrome patients were avoided by the intake of a sodium nitrate enriched beverage. Intake of about 8 mmol of sodium nitrate 30 min before exercise increased nitrate and nitrite plasma concentration, reduced the oxygen cost of the exercise, and prevented the increased pro-inflammatory mediators, PGs and other oxylipin plasma concentration produced by acute exercise. These effects of nitrate intake on the oxygen cost of exercise and on oxylipin production were mediated by the first reduction of nitrate to nitrite in the oral cavity and the posterior nitrite reduction to nitric oxide by nitrite reductase enzymes in the vasculature or other tissues. These processes ensure enough NO generation to produce nitrosative modification of target proteins acting as signaling molecules to induce a wide spectrum of biological responses [26,48,49]. We found that metabolic syndrome patients quickly reduce nitrate to nitrite at the millimolar level in the oral cavity, as an equilibrium between nitrate and nitrite concentration is attained after rinsing a high nitrate concentration for 2 min. Intake of 8 mmol sodium nitrate significantly increased the plasma concentration of both nitrate and nitrite, thus showing their assimilation. Dietary nitrate consumption in a single dose elevates plasma nitrite by about 100–200 nM [50,51], but prolonged supplementation does not induce a further increase [50,51]. The plasma ratio between nitrite and nitrate concentration is 100 times lower than in the oral cavity. This probably reflects a nitrite transformation back to nitrate, to nitric oxide, or both, in the stomach, vasculature, skeletal muscle, heart, and other tissues by several mechanisms including non-enzymatic disproportionation at low pH and enzyme-dependent reduction by nitrite reductase enzymes [52]. Nitrate intake in diabetes and metabolic syndrome patients has been pointed out to ameliorate insulin sensitivity and vascular function [53,54].

The effects of nitrate on reducing the energy cost of exercise in well-trained athletes are well documented [25,26,28]. It has been hypothesized that dietary nitrate could induce different carbohydrate or lipid substrate utilization during exercise [55], favoring the more efficient use of carbohydrates as opposed to lipids, but we observed no changes in the respiratory exchange ratio during exercise attributable to sodium nitrate intake. Another hypothesis could be a reduction in the ATP cost of force production by enhancing mitochondrial efficiency to produce ATP [56]. Larsen et al. [27] observed improved oxidative phosphorylation of the mitochondria isolated from the vastus lateralis muscle in subjects supplemented with sodium nitrate, suggesting reduced proton leakage and uncoupled respiration. Furthermore, increased mitochondrial P/O ratio (the amount of ATP produced/oxygen used) following nitrate supplementation has been correlated with a reduction in whole body oxygen uptake during exercise [27]. Nitrite could accept the electrons in the mitochondrial respiratory chain, producing nitric oxide [48] and contributing to the reduction of the oxygen cost of ATP production. In addition, oxygen is also a substrate of oxygenase enzymes such as COXs, LOXs, NADPH oxidases, xanthine oxidase, and hemo-oxygenases. Since these enzymes can also operate as mammalian nitrite reductases [48], this would also reduce oxygen consumption during exercise. In this respect, nitrite has been demonstrated to attenuate NADPH oxidase-derived superoxide generation in activated macrophages and human monocytes via a nitric oxide-dependent mechanism [29]. We contribute to this knowledge by reporting that dietary nitrate intake prevented increased plasma concentration of PGE1, PGE2 and 16-HPAL, probably as a direct effect of nitrite in the partial reduction of oxygen by COXs and CYP450. In fact, nitrate intake also significantly reduced the post-exercise production rate of 5-HETE and 15-HETE by PBMCs, and of 5-HETE by neutrophils. This reinforces the possible effect of nitrate intake on the reduced operation of COXs and heme-proteins such as CYP450.

## 5. Conclusions

**Figure 3:**
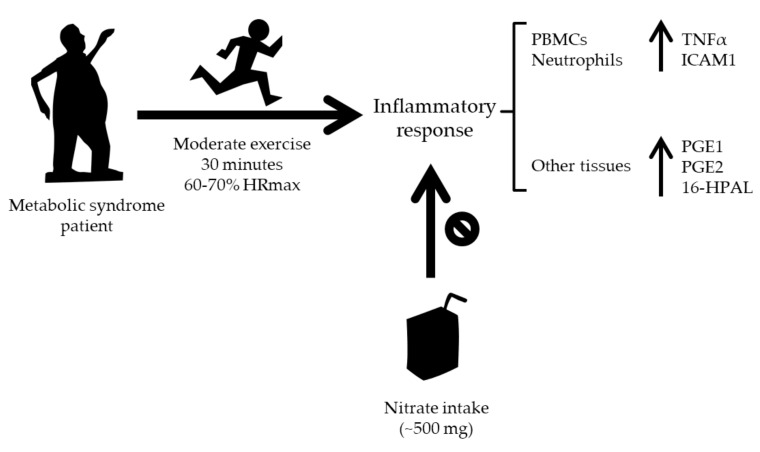
Graphical scheme summarizing the main findings of the article. 16-HPAL: 16-hydroxypalmitic acid; HRmax: maximal heart rate; ICAM1: intercellular adhesion molecule 1; PBMCs: peripheral blood mononuclear cells; PGE1: prostaglandin E1; PGE2: prostaglandin E2; TNFα: tumor necrosis factor α.

Aerobic exercise of moderate intensity and duration (30 min at 60–70% maximal heart rate) produced a mild systemic pro-inflammatory environment 30 min after exercise in metabolic syndrome patients, indicated by increased plasma concentration of the pro-inflammatory mediators TNFα and ICAM1, and also by an increased plasma concentration of the prostaglandins PGE1 and PGE2 and the newly detected hydroxyl saturated fatty acid 16-HPAL, probably reflecting an activated operation of enzymes involved in inflammation processes such as COXs and CYP450. PBMCs and neutrophils could be responsible for the basal plasma concentration of TNFα and IL6 but not for the plasma concentration of oxylipins, thus indicating that the changes induced by exercise in the plasma concentration of pro-inflammatory molecules and oxylipins are due to production by other tissues, such as contracting muscle. Intake of sodium nitrate 30 min before exercise increased the concentration of nitrate and nitrite in the oral cavity and plasma, reduced the oxygen cost of exercise, prevented the enhancing effects of acute exercise on pro-inflammatory mediators production and on PGs and other oxylipin plasma concentration, and reduced the capabilities of PBMCs and neutrophils to produce oxylipins. The main findings of this research are summarized in Figure 3. A practical application resulting from the present study could be the recommendation to ingest nitrate-rich foods or nitrate-enriched beverages before practicing exercise, which could contribute to the attenuation of the inflammatory response associated to exercise in patients with metabolic syndrome.

## Figures and Tables

**Table 1:** Individual anthropometric and clinical characteristics of subjects.

Patient	Age (Years)	Weight (kg)	Height (m)	BMI (kg/m^2^)	BP Syst/Diast (mmHg)	WC (cm)	HDLC (mg/dL)	TG (mg/dL)	Glucose (mg/dL)
**1**	65	74.4	1.62	28,0	15.3/8.2	100	40	174	87
**2**	60	89.7	1.78	28,3	13.1/7.7	102	59	152	85
**3**	57	80.6	1.69	28.1	14.3/8.9	102	31	108	95
**4**	63	98.8	1.72	33.2	15.3/8.3	122	52	231	153
**5**	73	95.2	1.59	37.6	14.7/8.6	122	38	346	102
**6**	63	118.7	1.73	39.3	14.4/8.6	127	63	149	126
**7**	59	94.2	1.65	34.5	13.0/8.1	116	40	232	104
**8**	60	90.3	1.76	29.0	14.6/6.3	105	47	119	145
**9**	61	73.9	1.61	28.5	13.0/8.2	103	74	167	91
**10**	55	121.5	1.88	34.2	14.2/9.7	122	28	463	120
**11**	59	83.8	1.69	29.3	14.1/8.5	103	28	125	121
**12**	68	82.6	1.66	29.9	13.5/6.6	105	46	446	96
**13**	58	88.8	1.64	32.8	14.7/9.6	114	46	177	100
**14**	70	75.3	1.63	28.07	19.5/7.0	108	52	82	85

BMI: Body mass index; BP: blood pressure; Diast: diastolic; HDLC: high-density lipoprotein cholesterol; Syst: systolic; TG: triglycerides; WC: waist circumference.

**Table 2:** Parameters of an exercise test at 60–70% of maximal heart rate after intake of placebo or a nitrate-supplemented beverage.

Parameter	Placebo	Nitrate
Test duration (min)	30.3 ± 0.1	30.5 ± 0.1
Speed (km/h)	4.4 ± 0.3	4.4 ± 0.3
Heart Rate (Beats/min)	103 ± 4.7	97.0 ± 3.5 *
VO_2_ (mL/min)	1408 ± 79	1245 ± 73 *
VO_2_ (mL/kg min)	16.2 ± 1.0	14.4 ± 1.0 *
VCO_2_ (mL/min)	1259 ± 69	1110 ± 61 *
Energy Expenditure (kcal/min)	6.9 ± 0.4	6.1 ± 0.4 *
Total Energy Expenditure (kcal)	210 ± 12	187 ± 11 *
Energy Efficiency (kcal/km)	97.6 ± 9.7	85.7 ± 5.6 *
Respiratory exchange ratio	0.89 ± 0.01	0.89 ±0.006
Energy from lipids (%)	37.3 ± 3.6	38.3 ± 2.1

Results represent mean ± SEM. Normality test: Shapiro–Wilk. All parameters followed a normal distribution. Statistical analysis: paired sample *t*-test, (*) represents significant differences vs. placebo (*p* < 0.05).

**Table 3:** Effects of an exercise test and acute sodium nitrate supplementation on plasma nitrite and nitrate concentrations.

NOx	Placebo		Supplemented		
	Pre-Exercise	Post-Exercise	Pre- Exercise	Post-Exercise	ANOVA
Nitrate (µM)	54.5 ± 11.6	53.8 ± 14.1	26.2 ± 6.1	261 ± 17 *#	E, ExS
Nitrite (µM)	0.14 ± 0.01	0.12 ± 0.01	0.13 ± 0.02	0.24 ± 0.05 *#	E’, ExS’
Nitrite/Nitrate Ratio (×100)	0.36 ± 0.11	0.29 ± 0.06	0.57 ± 0.12	0.09 ± 0.1 *#	E

Results represent mean ± SEM. Normality test: Shapiro–Wilk. All parameters followed a normal distribution after transformation to logarithmic scale. Statistical analysis: Two-way ANOVA for repeated measures (nitrate and nitrite, logarithmic. Ratio, scalar). E represents the significant effect of exercise; ExS represents significant interaction of exercise and supplementation; * represents significant differences vs. Pre-exercise; # represents significant differences vs. Placebo (*p* < 0.05). E’ represents the significant effect of exercise; ExS’ represents significant interaction of exercise and supplementation (*p* < 0.1).

**Table 4:** Effect of exercise and nitrate supplementation on white blood cell counts and inflammation plasma markers.

Parameter	Placebo		Supplemented		
	Pre-Exercise	Post-Exercise	Pre-Exercise	Post-Exercise	ANOVA
Neutrophils (10^3^/mm^3^) ^a^	4.00 ± 0.58	4.78 ± 0.57	3.81 ± 0.29	3.89 ± 0.08	
PBMCs (10^3^/mm^3^) ^a^	3.78 ± 0.45	3.09 ± 0.50	3.76 ± 0.35	3.41 ± 0.55	
TNFα (pg/mL) ^b^	198 (283)	248 (332) *’	212 (158)	176 (137)	N/A
IL6 (pg/mL) ^a^	6.82 ± 0.64	6.85 ± 0.54	6.11 ± 0.20	6.54 ± 0.53	
ICAM1 (pg/mL) ^a^	298 ± 20	387 ± 31 *	300 ± 21	306 ± 30	E
C Reactive Protein (mg/dL) ^a^	0.30 ± 0.20	0.29 ± 0.20	0.21 ± 0.09	0.21 ± 0.09	

^a^ Results represent mean ± SEM; ^b^ Results represent median (interquartile range). Normality test: Shapiro–Wilk. TNF-α, non-normal distribution; remaining parameters, normal distribution. Statistical analysis: Multiple Wilcoxon test (TNFα); two-way ANOVA for repeated measures (remaining parameters). E represents the significant effect of exercise; * represents significant differences vs. Pre-exercise (*p* < 0.05); *’ represents significant differences vs. Pre-exercise (*p* < 0.1). N/A: Not applicable due to non-parametric distribution. ICAM1: Intercellular adhesion molecule 1; IL6: interleukin 6; PBMC: peripheral blood mononuclear cells; TNFα: tumor necrosis factor α.

**Table 5:** Effect of exercise and nitrate supplementation on plasma fatty acids and oxylipin concentrations.

Compound	Placebo		Supplemented		ANOVA
	Pre-Exercise	Post-Exercise	Pre-Exercise	Post-Exercise	
AA (nM) ^a^	1939 ± 174	2073 ± 142	1897 ± 367	1883 ± 312	
ETA (nM) ^a^	20.3 ± 2.4	24.0 ± 5.1	15.8 ± 3.0	18.6 ± 3.3	
5-HETE (nM) ^a^	31.5 ± 3.6	42.0 ± 6.1	30.2 ± 6.1	33.6 ± 5.0	
15-HETE (nM) ^a^	8.54 ± 1.07	9.14 ± 1.11	6.69 ± 1.20	7.22 ± 0.98	
20-HETE (nM) ^a^	2.35 ± 0.36	2.62 ± 0.58	2.37 ± 0.79	1.94 ± 0.65	
PGE2 (nM) ^b^	30.6 (13.3)	95 (157) *’	31.5 (17.7)	36.8 (26.0)	N/A
EPA (nM) ^a^	453 ± 80	450 ± 45	342 ± 90	435 ± 113	
15-HEPE (nM) ^a^	6.02 ± 0.40	8.92 ± 1.41	4.46 ± 0.76	5.12 ± 0.59 #	E, S
DHA (nM) ^a^	5263 ± 842	4832 ± 632	5153 ± 682	4216 ± 1045	
17-DoHE (nM) ^a^	13.3 ± 2.1	15.6 ± 2.0	11.4 ± 1.4	9.83 ± 1.12	
RvD2 (nM) ^a^	0.38 ± 0.09	0.29 ± 0.10	0.39 ± 0.15	0.40 ± 0.13	
16-HPAL (nM) ^a^	29.6 ± 2.8	66.2 ± 10.0 *	35.7 ± 4.3	37.3 ± 6.5 #’	E, ExS
PGE1 (nM) ^a^	36.3 ± 5.7	50.4 ± 8.9 *	31.2 ± 2.7	31.9 ± 1.1	E

^a^ Results represent mean ± SEM, ^b^ Results represent median (interquartile range). Normality test: Shapiro-Wilk. AA, ETA, 15-HETE, 20-HETE, EPA, 17-DoHE and PGE1, normal distribution; 5-HETE, 15-HEPE, DHA, RvD2 and 16-HPAL, normal distribution after transformation to a logarithmic scale; PGE2, non-normal distribution. Statistical analysis: Multiple Wilcoxon test (PGE2); Two-way ANOVA for repeated measures in scalar or logarithmic scale (remaining parameters). E represents the significant effect of exercise; ExS represents significant interaction of exercise and supplementation; N/A represent the ANOVA column does not apply due to the application of non-parametric tests; * represents significant differences vs. Pre-exercise (*p* < 0.05); *’ represents significant differences vs. Pre-exercise (*p* < 0.1), # represents significant differences vs. Placebo (*p* < 0.05), #’ represents significant differences vs. Placebo (*p* < 0.1). AA: 5,8,11,14-Eicosatetraenoic acid (Arachidonic acid); DHA: 4,7,10,13,16,19-docosahexaenoic acid; 17-DoHE: 17-Hydroxy-4,7,10,13,16,19-docosahexaenoic acid; EPA: 5,8,11,14,17-eicosapentenoic acid; ETA: 8,11,14,17-eicosatetraenoic acid; 15-HEPE: 15-Hydroxy-5,8,11,13,17-Eicosapentaenoic acid; 5-HETE: 5-Hydroxyi-6,8,11,14-Eicosatetraenoic acid; 15-HETE: 15-Hydroxy-5,8,11,13-Eicosatetraenoic acid; 20-HETE: 20-Hydroxy-5,8,11,13-Eicosatetraenoic acid; 16-HPAL: 16-Hydroxy-hexadecanoic acid; PGE1: Prostaglandin E1; PGE2: Prostaglandin E2; RvD2: Resolvin D2.

**Table 6:** In vitro production of cytokines, fatty acids and oxylipins by peripheral blood mononuclear cells (PBMCs) and neutrophils isolated in basal conditions (pre-exercise, no supplementation) and cultured for 2 h at 37 °C.

Compound	PBMCs	NEUTROPHILS
TNFα (pg/mL) ^a^	301 ± 38	127 ± 26 *
IL6 (pg/mL) ^b^	92.9 (84.9)	24.9 (21.2) *
AA (nM) ^b^	12.1 (6.5)	6.16 (14.9)
ETA (nM) ^b^	0.040 (0.095)	0.016 (0.154)
5-HETE (nM) ^b^	1.85 (2.66)	0.120 (0.567) *
15-HETE (nM) ^b^	1.29 (1.98)	0.13 (1.30) *
PGE2 (nM) ^b^	0.607 (0.82)	4.52 (4.55) *
EPA (nM) ^b^	3.41 (4.07)	4.25 (41.4)
15-HEPE (nM) ^b^	0.17 (1.21)	0.000 (0.049) *
DHA (nM) ^b^	4.95 (3.14)	1.03 (1.49) *
17-DoHE (nM) ^b^	1.60 (9.64)	0.000 (0.006) *
16-HPAL (nM) ^b^	24.5 (15.7)	3.10 (4.98) *
PGE1 (nM) ^b^	0.030 (0.052)	0.000 (0.031) *

The production of cytokines, oxylipins or fatty acids is expressed as the concentration of these compounds in the culture medium after 2 h of PBMCs or neutrophil incubation at the same cell counts as in blood. ^a^ Results represent mean ± SEM. ^b^ Results represent median (interquartile range). Normality test: Shapiro–Wilk. TNFα, normal distribution; remaining parameters, non-normal distribution. Statistical analysis: Paired-samples t-test for normally distributed parameters, Wilcoxon test for not normally distributed parameters. (*) represents significant differences vs. PBMCs (*p* < 0.05). AA: 5,8,11,14-Eicosatetraenoic acid (Arachidonic acid); DHA: 4,7,10,13,16,19-docosahexaenoic acid; 17-DoHE: 17-Hydroxy-4,7,10,13,16,19-docosahexaenoic acid; ETA: 8,11,14,17-eicosatetraenoic acid; EPA: 5,8,11,14,17-eicosapentenoic acid; 15-HEPE: 15-Hydroxy-5,8,11,13,17-Eicosapentaenoic acid; 5-HETE: 5-Hydroxyi-6,8,11,14-Eicosatetraenoic acid; 15-HETE: 15-Hydroxy-5,8,11,13-Eicosatetraenoic acid; 16-HPAL: 16-Hydroxy-hexadecanoic acid PGE1: Prostaglandin E1; PGE2: Prostaglandin E2.
